# Environmental Monitoring of Methane with Quartz-Enhanced Photoacoustic Spectroscopy Exploiting an Electronic Hygrometer to Compensate the H_2_O Influence on the Sensor Signal

**DOI:** 10.3390/s20102935

**Published:** 2020-05-22

**Authors:** Arianna Elefante, Giansergio Menduni, Hubert Rossmadl, Verena Mackowiak, Marilena Giglio, Angelo Sampaolo, Pietro Patimisco, Vittorio M. N. Passaro, Vincenzo Spagnolo

**Affiliations:** 1 PolySense Lab-Physics Department, University and Polytechnic of Bari, CNR-IFN, 70126 Bari, Italy; Arianna.elefante@uniba.it (A.E.); giansergio.menduni@poliba.it (G.M.); marilena.giglio@poliba.it (M.G.); angelo.sampaolo@poliba.it (A.S.); pietro.patimisco@uniba.it (P.P.); 2 Photonics Research Group, Department of Electrical and Information Engineering, Polytechnic University of Bari, 70126 Bari, Italy; vittorio.passaro@poliba.it; 3 Thorlabs GmbH, Münchner Weg 1, 85232 Bergkirchen, Germany; hrossmadl@thorlabs.com (H.R.); vmackowiak@thorlabs.com (V.M.)

**Keywords:** quartz-enhanced photoacoustic spectroscopy, methane, hygrometer, relaxation promoter, environmental monitoring

## Abstract

A dual-gas sensor based on the combination of a quartz-enhanced photoacoustic spectroscopy (QEPAS) sensor and an electronic hygrometer was realized for the simultaneous detection of methane (CH_4_) and water vapor (H_2_O) in air. The QEPAS sensor employed an interband cascade laser operating at 3.34 μm capable of targeting a CH_4_ absorption line at 2988.8 cm^−1^ and a water line at 2988.6 cm^−1^. Water vapor was measured with both the electronic hygrometer and the QEPAS sensor for comparison. The measurement accuracy provided by the hygrometer enabled the adjustment of methane QEPAS signal with respect to the water vapor concentration to retrieve the actual CH_4_ concentration. The sensor was tested by performing prolonged measurements of CH_4_ and H_2_O over 60 h to demonstrate the effectiveness of this approach for environmental monitoring applications.

## 1. Introduction

Methane (CH_4_) is one of the main anthropogenic greenhouse gases in the atmosphere. Its concentration has increased up to 1.87 ppm, starting from a value of 715 ppb in preindustrial times [1]. Due to the effect of CH_4_ on global warming and climate change, methane detection is mandatory to monitor variations in atmospheric concentration as well as identify its main sources. These objectives can be fulfilled by real-time and in situ measurements of CH_4_ concentration in the atmosphere. Thus, a methane sensor must guarantee the following: (i) high sensitivity in the sub-part-per-million range; (ii) high selectivity to discriminate the CH_4_ signal from other gas components in the atmosphere; and (iii) fast response time to track any variation in concentration. Moreover, robustness, compactness, and insensibility to environmental external noise are required for in-field operation. Various types of sensors have been developed to detect methane in the atmosphere, showing different advantages and disadvantages in terms of selectivity, sensitivity, response time, robustness, and stability for prolonged measurements. A common issue to be addressed is the presence of water in air, the change in humidity of which affects the performance of sensors. Semiconductor sensors based on metal oxide film are robust and long-lived but not suitable for in-field detection of methane. They require high power consumption to keep the metal oxide at the 100–500 °C temperature range, are sensible to variations in temperature and humidity, and suffer from poor selectivity. In [2], a CH_4_ semiconductor sensor was tested with a measurement of the lab air for 31 days, showing an accuracy of the order of 0.8–2.7 ppm, which precluded its application for atmospheric CH_4_ monitoring. Nondispersive infrared spectroscopy (NDIR) sensors do not have the sensitivity level required for environmental application. In addition, they suffer from spectral interference by water, and the use of optical filters requires a dry ambience because water condensation causes a variation in the transmittance efficiency. Laser absorption spectroscopy (LAS) employs a laser as an excitation source to improve detection selectivity, overcoming the need for optical filters used in NDIR. It offers high detection sensitivity thanks to the exploitation of multipass gas cells to increase the molecule absorption path length. In [3], a multipass cell was used in a LAS-based sensor to detect atmospheric methane, reaching a detection sensitivity of 100 ppb. Measurements of CH_4_ in ambient air were performed for two days using a dryer and a particle filter to remove humidity. A cavity ring-down spectroscopy (CRDS) sensor was developed by Picarro for the detection of CH_4_, H_2_O, and CO_2_ in the atmosphere, reaching a CH_4_ minimum detection limit of less than 1 ppb for an integration time of 5 s [4]. In [5], a comparison of portable devices for the detection of methane for soil research was reported. The performance of LAS and Fourier transform infrared spectroscopy (FTIR) sensors were tested, showing detection limits of 0.01 ppm with 10 s response time and 0.053 ppm with less than 120 s response time, respectively. A review and comparison of optical sensors for CH_4_ detection is reported in [6], even though most of the sensors have not been tested with prolonged measurements of CH_4_ in ambient air. Among LAS sensors [7,8,9], QEPAS sensors were found to fulfil all the needed requirements, providing highly sensitive measurements of CH_4_ [10,11,12]. QEPAS is based on the absorption of modulated laser light by the target gas. The laser beam is focused between the prongs of a quartz tuning fork (QTF) at one of the antinode points of the QTF vibrational mode and is modulated at the associated resonance frequency or at one of its subharmonics. The energy of the excited roto-vibrational states is released via inelastic collisions among the surrounding molecules, generating a pressure wave. The pressure wave is detected by the quartz tuning fork, acting as a transducer of the prongs’ mechanical deflection induced by the pressure wave, into an electrical signal thanks to the piezoelectricity of the quartz [13]. The QTF is acoustically coupled with microresonator tubes to amplify the sound wave [14]. The generation of the acoustic wave, and consequently of the QEPAS signal, depends on the relaxation rate of the excited molecules’ vibrational energy into the kinetic energy (translation) of the surrounding molecules (VT relaxation). This effect has been investigated in several studies [15,16], and it has become particularly relevant for detection of gas species with slow VT relaxation rates, such as CH_4_. In the latter case, a laser modulation frequency lower than the effective analyte relaxation rate in the gas matrix can be selected in order to allow a complete release of the absorbed energy between consecutive optical excitations. This guarantees a highly efficient sound wave generation. The development of custom QTFs with resonance frequencies lower than 20 kHz [17] was aimed to address this issue. However, the molecule relaxation rate depends on the gas matrix, and a variation in the matrix composition can affect the QEPAS signal, particularly in the case of slow relaxing gases. 

Once the gas target molecules are excited, they can relax through different channels via collisions with any kind of molecule composing the mixture. The relaxation rate of the target molecule in a matrix is then provided by the sum of the relaxation rates characterizing every possible energy transfer pathway, weighted by the concentration of each species in the mixture. In particular, for environmental monitoring application, CH_4_ is detected in a standard air-like matrix containing water vapor in the concentration range of a few percentage points. The required CH_4_ and H_2_O concentration range for environmental applications are 0.1–1000 ppm and from 100 ppm to 3%, respectively. The influence of water on the performance of QEPAS methane sensors has been investigated in several studies [18,19]. The water vapor molecules act as promoters for the VT relaxation processes of CH_4_. As a result, a variation of water concentration in air causes a variation in the QEPAS methane signal not related to a change in the CH_4_ concentration. In order to guarantee reliable CH_4_ concentration measurement, two sensor configurations can be implemented. The first one involves adding a humidifier in series to the gas delivery line to fix the H_2_O concentration [20,21,22] and calibrate the sensor. The benefit is enhancement of the CH_4_ signal due to the presence of constant H_2_O concentration, resulting in higher CH_4_ detection sensitivity. On the other hand, a periodic check on the humidifier quality is mandatory. For example, a constant humidity of the gas mixture can be achieved by flowing the gas through a Nafion membrane humidifier immersed in a temperature-controlled water bath, which requires periodic refilling. The second approach consists of measuring the H_2_O and CH_4_ concentrations simultaneously using a multigas detection scheme and properly adjusting the CH_4_ QEPAS signal with respect to the H_2_O signal. In this case, either multiple laser sources operating simultaneously can be employed or a single laser source whose spectral range covers both water and methane absorption lines [23,24]. In [24], the atmospheric detection of N_2_O, CH_4_, and H_2_O was achieved using a single QCL whose spectral range covers absorption lines of all three gases. In [4], atmospheric measurements of CH_4_, CO_2_, and H_2_O were performed using a cavity ring-down analyzer consisting of two lasers alternatively selected using an optical switch. In [25], a QEPAS sensor based on a frequency division multiplexing scheme with a single QTF and two laser sources was implemented to simultaneously detect H_2_O and CH_4_. In that case, the water signal was used to compensate the influence of H_2_O on the CH_4_ signal. However, using a single laser source does not allow a simultaneous detection of both gas species and an instantaneous calibration; on the other hand, using multilaser sources increases the complexity of the experimental apparatus, making the sensor less suitable for in-field applications.

In this work, we demonstrated that for the environmental monitoring of CH_4_, a methane QEPAS sensor can be used in combination with an electronic hygrometer monitoring the variation of water vapor in the air. For comparison, the water signal was also detected using the same QEPAS sensor, targeting a water absorption line nearby the methane one. The accuracy and precision of the H_2_O concentration measurements provided by the hygrometer allows compensating the water influence on CH_4_ QEPAS signal. 

## 2. Experimental Setup

**Figure 1:**
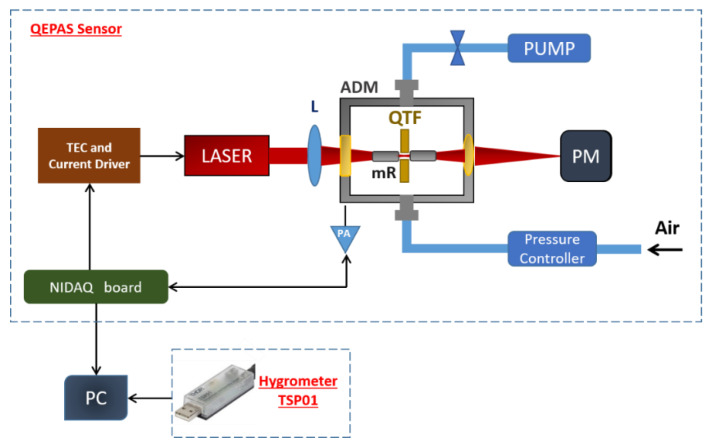
Schematic of the sensor setup for methane and water vapor detection including a quartz-enhanced photoacoustic spectroscopy (QEPAS) module and a hygrometer. ADM, acoustic detection module; QTF, quartz tuning fork; mR, microresonators; L, focusing lens; PM, power meter; PA, preamplifier; NIDAQ board, National Instruments data acquisition board; PC, personal computer.

A schematic of the experimental apparatus used in this work is shown in Figure 1 and consists of a combination of two sensors: a QEPAS-based sensor for the detection of CH_4_ and H_2_O and a temperature and humidity sensor for monitoring the H_2_O concentration in air. 

The QEPAS sensor for the detection of CH_4_ and H_2_O is shown in the upper part of Figure 1. The light source was an interband cascade laser (ICL) emitting at 3.345 µm, capable of targeting two nearby CH_4_ and H_2_O absorption lines. The laser beam was focused into an acoustic detection module (ADM) using lens with 40 mm focal length. The ADM contained a custom quartz tuning fork with fundamental resonance frequency f_0_ = 12456.9 Hz at 200 Torr. The QTF was acoustically coupled with two 12.4 mm long tubes with internal diameter of 1.6 mm to amplify the acoustic wave and enhance the QEPAS signal [17]. The laser beam was refocused on the sensitive element of a power meter set behind the ADM. The ICL current and temperature were controlled using a Benchtop Laser Diode/TEC Controller (Model ITC4002–Thorlabs, Newton, MA, USA). At the laser operating temperature of 25 °C, the optical power measured by the power meter was 12.5 mW. QEPAS measurements were performed using wavelength modulation with 2f-detection; a sinusoidal modulation was applied to the laser current at half of the QTF resonance frequency, and the QTF response was detected at f_0_ using a digital lock-in amplifier. QEPAS spectral scans were obtained by sweeping the laser current by a 4.4 mHz ramp. A National Instrument data acquisition board together with a dedicated LabVIEW-based software was used to feed the modulation and ramp to the ICL current driver and to acquire and demodulate the QTF signal. The pressure and flow of the sample gas inside the ADM were controlled and fixed using a system composed of a pressure controller, a needle valve, and a pump. The temperature and relative humidity of the air in laboratory environment close to the QEPAS sensor were also monitored using the an hygrometer (model TSP01–Thorlabs, Newton, MA, USA) with the dimensions of a USB stick. TSP01 was directly connected to the computer.

## 3. Measurements of CH_4_ and H_2_O in Air

**Figure 2:**
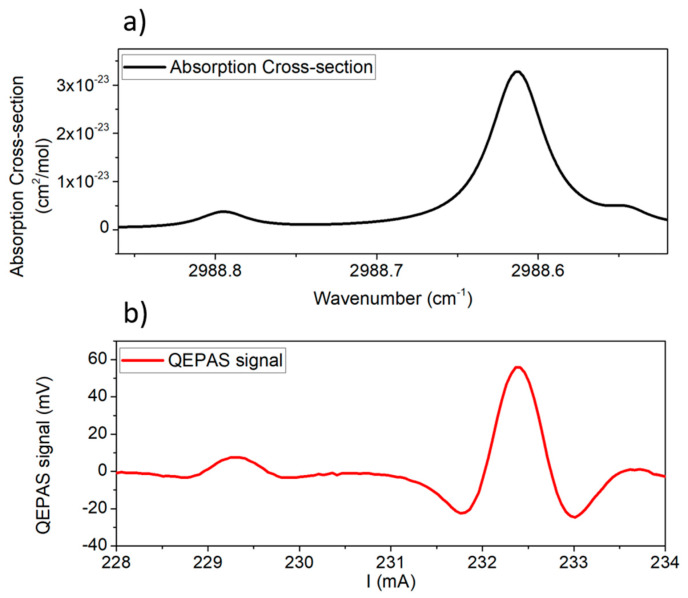
(**a**) Absorption spectrum of standard air simulated with HITRAN database at 200 Torr and room temperature; (**b**) QEPAS spectral scan of laboratory ambient air at 200 Torr.

Figure 2 shows comparison between the absorption cross section of standard air at 200 Torr simulated using the HITRAN database [26] (Figure 2a) and a representative QEPAS scan of the laboratory air (Figure 2b).

**Figure 3:**
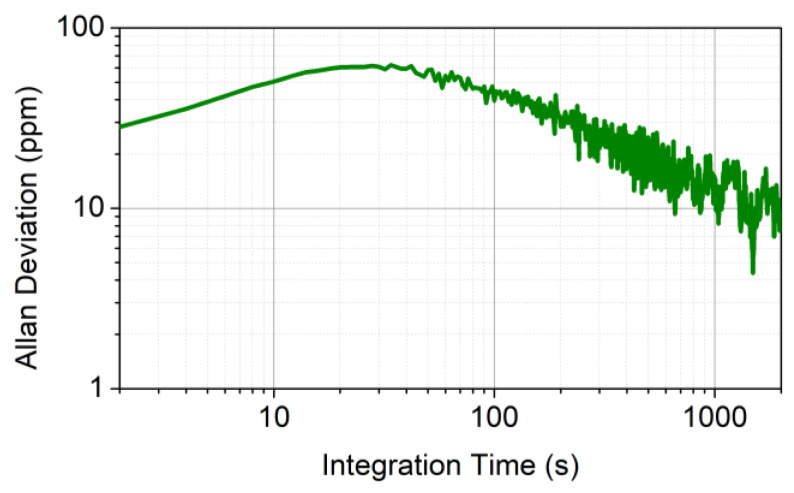
Allan deviation in ppm of the absolute humidity calculated using Equation (1).

From comparison, the highest peak at 232.35 mA clearly corresponds to the water absorption line, while the lower peak at 229.25 mA corresponds to the methane line. A preliminary calibration of the QEPAS sensor for the detection of CH_4_ in dry N_2_ was performed. The sensor was calibrated by acquiring the QEPAS signal of the CH_4_ absorption line at 2988.8 cm^−1^ for different concentrations of CH_4_. The mixtures were obtained starting from a certified concentration of 45 ppm of CH_4_, which was diluted in pure N_2_ by means of a gas mixer. By linearly fitting the peak values as a function of the CH_4_ concentration, the calibration curve y = (1.07 mV/ppm) · x was obtained with an R^2^ = 0.999, confirming the linearity of the sensor response with respect to the CH_4_ concentration. A minimum detection limit of ~180 ppb was achieved for a 1σ noise of 0.20 mV at 200 ms integration time. We performed an Allan variance analysis [27] of the hygrometer TSP01 signal to study the long-term stability of the absolute humidity measurement. The TSP01 sensor was closed in a climate chamber to fix the temperature and the relative humidity of surrounding air at 27 °C and 40%, respectively. The TSP01 signal was acquired for ~4 h with an integration time of 2 s. The absolute humidity is the total mass of water vapor present in a certain volume or mass of air. It gives a measurement of the concentration of water vapor in air. The relative humidity is the ratio between the amount of water vapor in air and the amount of water vapor that would saturate the air at the same temperature and pressure. The H_2_O QEPAS signal is a measurement of the absolute humidity; TSP01 measures the relative humidity and the temperature of air. Both temperature (*T*) and relative humidity (*RH*) values were used to calculate the absolute humidity (*AH*) of the air sample, in ppm using the following equations [28,29]:

(1) $$AH=10^{6}*\frac{P_{w}}{P-P_{w}},\;\;\;P_{w}=P_{ws}*\frac{RH}{100},\;\;\;P_{ws}=6.11*\exp\left(\frac{17.7*T}{T+243.57\;{}^{\circ}C}\right)$$

where *P* is the ambient pressure (760 Torr), *P_w_* is the water vapor pressure, and *P_ws_* is the saturated water vapor pressure. In Figure 3, the Allan deviation of the absolute humidity signal (calculated with Equation (1) using the relative humidity and the temperature measured by TSP01) is shown as a function of the signal integration time.

The Allan deviation slightly increased from 2 to 20 s and then followed the √t dependence expected, where the dominant noise source was the flicker noise. The accuracy of temperature and relative humidity measurements were 0.5 °C and 2%, respectively, as reported in the datasheet of the instrument. The precision of TSP01, evaluated experimentally with prolonged measurements of T and RH at fixed condition, was 0.01 °C for temperature and 0.1% for relative humidity. These values determined an accuracy and a precision on the calculated absolute humidity of ~350 and ~30 ppm, respectively, estimated using the error propagation for Equation (1).

**Figure 4:**
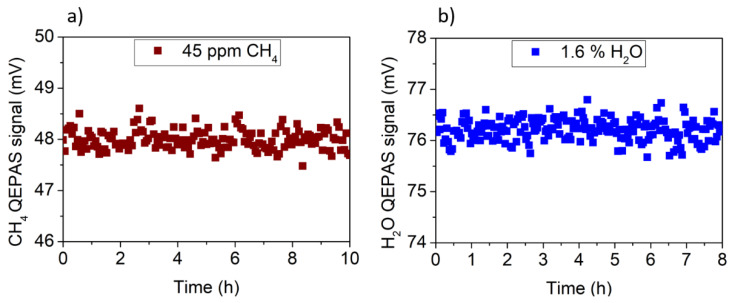
(**a**) Methane QEPAS peak signal as a function of time when a gas mixture of 45 ppm of dry CH_4_ in N_2_ was flowing in the sensor; (**b**) H_2_O QEPAS peak signal as a function of time when water concentration in the gas line was fixed to 1.6% using a PermSelect humidifier.

An investigation of the long-term stability of the methane peak values was performed when no water vapor was in the gas line. With this aim, a 10 h long measurement of fixed 45 ppm CH_4_ concentration in dry N_2_ was carried out. The measurements were performed by acquiring QEPAS spectral scans of the CH_4_ absorption line with a 200 ms integration time and by extracting the peak value from each scan. Similarly, an 8 h long measurement of fixed concentration of H_2_O was performed to test the long-term stability of the H_2_O QEPAS peak values. A PermSelect humidifier was inserted in the gas line upstream the ADM to keep water concentration fixed to 1.6%. The CH_4_ and the H_2_O QEPAS peak values are reported in Figure 4 as a function of time.

**Figure 5:**
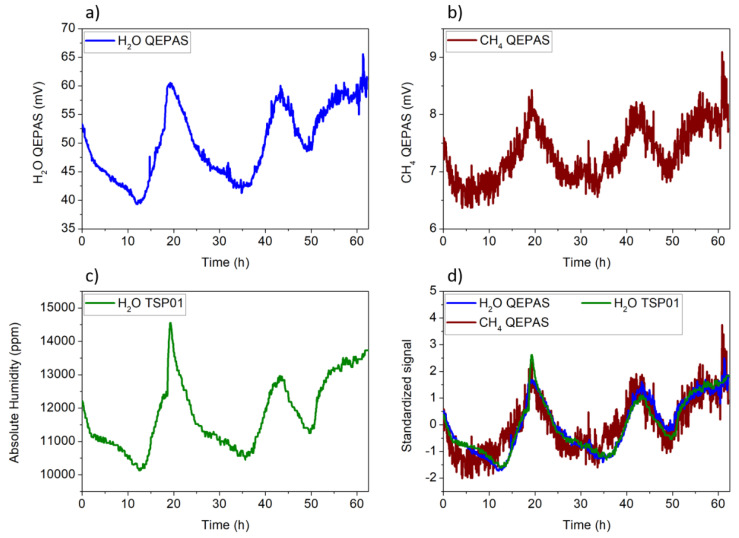
QEPAS peak signal vs. time for (**a**) water vapor and (**b**) methane; (**c**) absolute humidity calculated from the temperature and relative humidity detected with TSP01 and using Equation (1); (**d**) CH_4_ QEPAS, H_2_O QEPAS, and absolute humidity signals standardized with respect to their mean and standard deviation values.

Both the CH_4_ and H_2_O QEPAS signals had no appreciable drifts during 10 h of continuous measurement. The 1σ value of fluctuations was 0.20 mV for CH_4_ and 0.22 mV for H_2_O, confirming the long-term stability of the sensor when both gases are detected separately. Once the sensor was calibrated, the CH_4_ and H_2_O concentrations in laboratory ambient air were continuously monitored for 62 h over a weekend. The CH_4_ signal was measured using the QEPAS sensor, while the H_2_O signal was acquired using both the QEPAS sensor and the hygrometer TSP01. ICL wavelength shifts can affect the QEPAS measurement. This is expected when the laser line is fixed to the CH_4_ absorption peak without a line-locking feedback system. To avoid this issue, QEPAS spectral scans were acquired by setting the temperature of the ICL to 25 °C and scanning the laser current in the range 228–234 mA, to detect both CH_4_ and H_2_O (see Figure 2b) absorption lines. The pressure and the flow of the sample air flushed through the ADM were set to 200 Torr and 25 standard cubic centimeter per minute (sccm), respectively. For each spectral scan, the QEPAS peak signals of H_2_O and CH_4_ absorption features were extracted and are plotted as a function of time in Figure 5a,b, respectively. The time interval between two consecutive peaks of the same gas species was 3.8 min. Simultaneously, the TSP01 sensor was placed close to the QEPAS sensor to acquire the temperature and the relative humidity of the laboratory ambient air. The temperature and relative humidity excursion intervals recorded during 62 h of sensor operation were 24–28 °C and 32–44%, respectively. The absolute humidity is plotted in Figure 5c as a function of time.

Figure 5d reports the standardized CH_4_ QEPAS, H_2_O QEPAS, and the absolute humidity signals, overlapped on the same *x*-axis; each standardized signal was obtained by subtracting from the original signal its mean and dividing the difference by the standard deviation. The three signals exhibited the same trend as a function of time. Thus, the QEPAS sensor and the electronic hygrometer detected the same water vapor variations in ambient air, which in turn affected the CH_4_ signal. Shifts of QTF resonance frequency as well as variations in the Q factor can affect QEPAS measurements. Indeed, the resonance frequency of the QTF determines the modulation frequency applied to the laser current, while the QEPAS signal is proportional to the Q factor itself. At the beginning and at the end of the 62 h long series of measurements, the QTF resonance curve was acquired, and no appreciable shifts in the frequency or sensible variations of the Q factor were measured.

**Figure 6:**
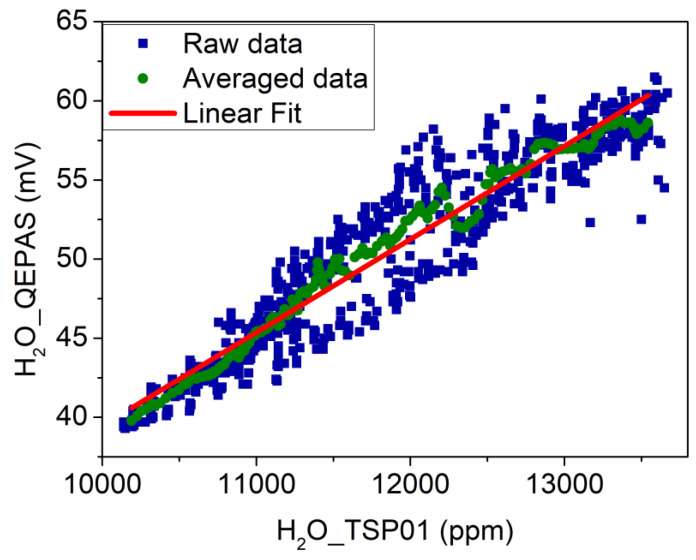
H_2_O QEPAS signal plotted as a function of the absolute humidity (blue squares). Averaged dataset obtained with a moving average on 20 points (green circles); linear fit performed on the averaged dataset (red solid line).

As a first step, the absolute humidity values measured by TSP01 can be used to calibrate the QEPAS sensor for water vapor detection. In Figure 6, the H_2_O QEPAS signal is plotted as a function of the absolute humidity (blue squares). 

The deviations of the H_2_O QEPAS signals with respect to hygrometer measurements can be mainly ascribed to a different precision of the two acquisition techniques and to the fact that the QTF is located in the gas cell, while the hygrometer is placed in the outside environment. The most immediate technique for smoothing signals consisting of equidistant points is the moving average. With a fixed subset size, the first element of the moving average is obtained by taking the average of the initial fixed subset of the number series. Then, the subset is modified by excluding the first datum of the series and including the next value in the subset. A LabVIEW-based software was implemented to perform a moving average and at the same time establish the optimized size of subsets. Starting from datasets with two points, a linear fit was performed on the obtained “smoothed” signal, and the R^2^ value was extracted. Then, the subset size was increased, and R^2^ values were plotted as a function of the subset size. We observed that R^2^ value rapidly rose as the subset size increased until a plateau value of 0.99 was reached. This condition was obtained when the subset size was 20. Figure 6 shows the averaged dataset when the subset size was 20 (green circles) and the best linear fit (red line), which returned a slope of *k*_1_ = 5.9 μV/ppm and a negligible intercept. This curve can be used as a calibration curve to convert the H_2_O QEPAS signal into water vapor concentration. 

**Figure 7:**
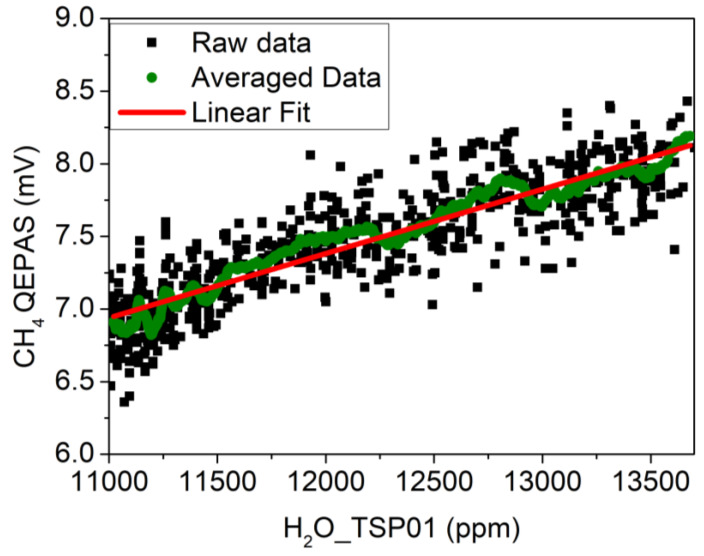
CH_4_ QEPAS signal in standard laboratory air plotted as a function of the absolute humidity of the air measured with the TSP01 sensor (black squares). Dataset obtained with a moving average on 25 points (green circles); linear fit performed on the averaged dataset (red solid line).

Figure 5d shows the influence of H_2_O variations on the CH_4_ QEPAS signal due to water vapor acting as a relaxation promoter for methane. As the laboratory was closed without people inside for the entire duration of the measurement, the CH_4_ concentration can be assumed to be constant with Gaussian-distributed fluctuations. At atmospheric concentrations level, the CH_4_ QEPAS signal varies linearly with absolute humidity, as demonstrated in previous studies [25]. Thus, the CH_4_ QEPAS signal is plotted as a function of the absolute humidity in Figure 7 (black squares). 

**Figure 8:**
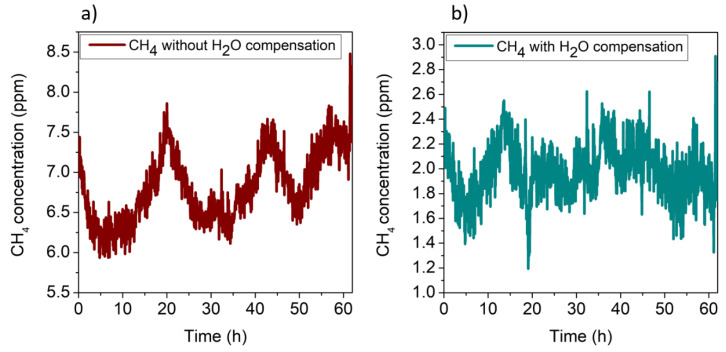
(**a**) QEPAS signal of methane in air without water compensation; (**b**) methane QEPAS signal after water compensation using the absolute humidity recorded by the hygrometer.

A moving average was implemented on the dataset with subset size of 25 (green circles). The corresponding calibration curve (red line) is the linear fit to averaged dataset, resulting in a slope of *m*_1_ = 0.4 μV/ppm, an intercept of *q*_1_ = 1.95 mV, and a R^2^ of 0.98. This calibration curve was used to compensate the influence of the H_2_O concentration in air on the CH_4_ signal using the following equation:

(2) $$\bar{CH_{4}}\;\left(mV\right)=\left[CH_{4}\_\_QEPAS\;\left(mV\right)-m1\left(\frac{mV}{ppm}\right)*H_{2}O\_\_TSP01\left(ppm\right)\right]$$

where $\bar{CH_{4}}$ is the signal obtained with water compensation, $CH_{4}\_\_QEPAS$ is the measured CH_4_ QEPAS signal, and $H_{2}O\_\_TSP01$ is the absolute humidity measured with the hygrometer. Figure 8 shows the comparison between the CH_4_ concentration detected by the QEPAS sensor without (Figure 8a) and with (Figure 8b) water compensation. 

**Figure 9:**
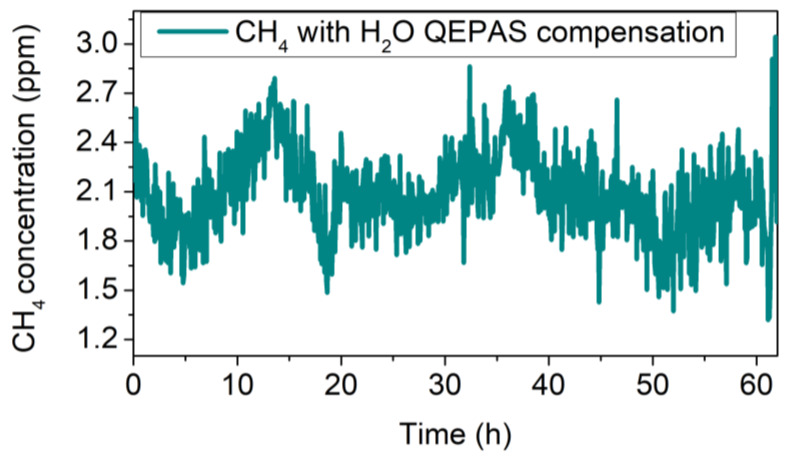
Methane QEPAS signal after water compensation using the H_2_O QEPAS signal.

In both cases, the calibration curve of CH_4_ in N_2_ (*y =* 1.07 mV/ppm *x*) was used to convert the *y*-axis from mV to ppm. It is worth noting that a mean value of CH_4_ concentration of 6.82 ppm with a standard deviation (1σ) of 0.44 ppm was estimated without water compensation (Figure 8a), which does not represent a reliable measurement of the atmospheric CH_4_ concentration. Conversely, the use of the electronic humidity sensor allowed correct calibration of the CH_4_ signal, resulting in a mean concentration value of 1.95 ± 0.25 ppm, significantly lower than the concentration estimated without water compensation and a noise comparable with that of the CH_4_ sensor calibrated using the CH_4_–N_2_ mixtures. For comparison, the H_2_O QEPAS signal was used for the correction of CH_4_ QEPAS measurement instead of TSP01. The results are shown in Figure 9. 

The corrected CH_4_ signal showed a trend similar to those obtained when TSP01 was used for CH_4_ signal compensation (see Figure 8b). A standard deviation of 0.27 ppm was measured, comparable with the 0.25 ppm value obtained using TSP01. As the use of the H_2_O QEPAS signal as methane signal compensation does not add any improvement, we have demonstrated that the use of the hygrometer is a valid alternative when the laser spectral range does not cover a H_2_O absorption line. 

**Figure 10:**
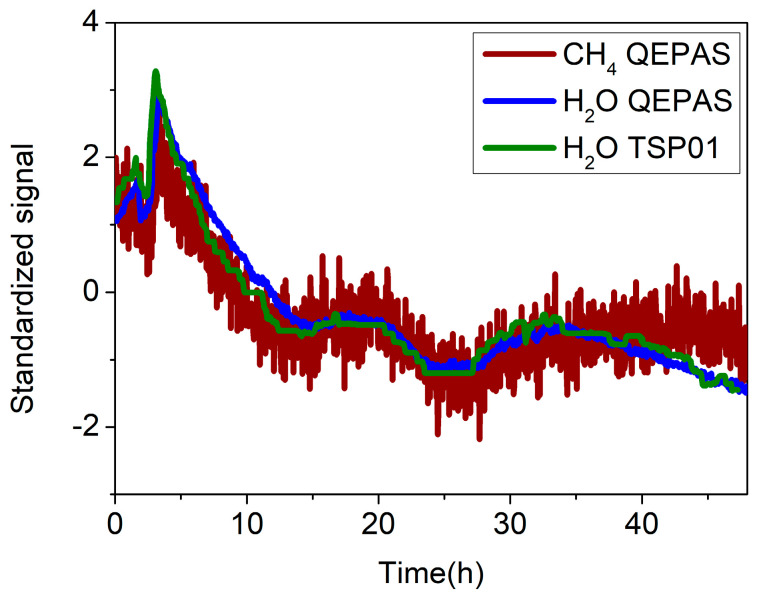
Standardized signals of CH_4_ (red line) and H_2_O (blue line) in laboratory air detected with the QEPAS sensor. Standardized absolute humidity detected with the TSP01 hygrometer (green line). Standardization was accomplished with respect to their mean and standard deviation values.

To verify the repeatability of the measurement, a second set of data was acquired for 48 h (the next weekend), and the same analysis was performed on this new dataset. Figure 10 shows the CH_4_ QEPAS signal (red line), the H_2_O QEPAS signal (blue line), and the H_2_O signal measured by the electronic hygrometer (green line) standardized to their mean and standard deviation values. Again, the influence of water on CH_4_ signal is clearly visible.

**Figure 11:**
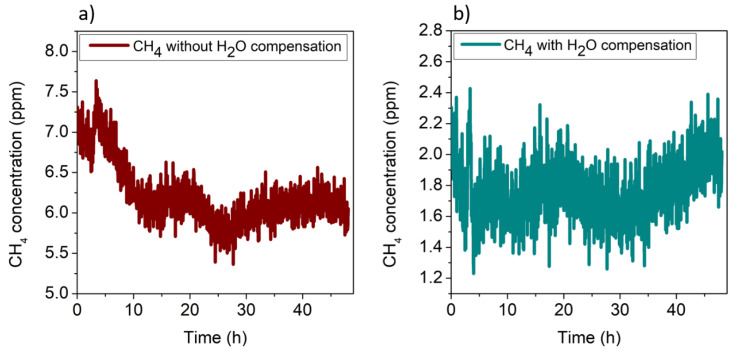
(**a**) QEPAS signal of the methane in laboratory air without water compensation; (**b**) methane QEPAS signal after the compensation with the absolute humidity recorded by the hygrometer.

As for the first dataset, the H_2_O and CH_4_ QEPAS signals as a function of the absolute humidity were averaged and linearly fitted; a slope of *m*_2_ = 0.4 μV/ppm was extracted, matching the value of *m*_1_, thus demonstrating the repeatability of the measurements. The CH_4_ QEPAS signals were corrected using the linear fit with slope *m*_2_ as calibration curve. The results without and with water calibration are shown in Figure 11a,b, respectively, where the *y*-axis has been converted in ppm using the calibration curve of CH_4_ in N_2_. A mean concentration value of methane in atmosphere of 1.76 ± 0.2 ppm was extracted, comparable with the previous estimation.

Although its sensitivity as well as response time is worse than H_2_O QEPAS detection, we have demonstrated that a hygrometer can be successfully used to compensate the CH_4_ QEPAS signal for a reliable detection in the atmosphere. Therefore, this approach represents a valid solution that can be easily extended for detection of all gas species with the QEPAS technique without the use of an additional laser source to target water vapor.

## 4. Conclusions

In this work, we have reported a QEPAS sensor for the detection of atmospheric CH_4_. The sensor exploits an electronic hygrometer to monitor the H_2_O concentration and compensate the influence of H_2_O on the CH_4_ signal. The sensor was calibrated by acquiring the QEPAS signal of the CH_4_ absorption line at 2988.8 cm^−1^ for different concentrations of CH_4_ in the range 1–45 ppm, with a minimum detection limit of 180 ppb at 200 ms integration time. The corresponding absolute humidity excursion interval recorded during the measurement was 1–1.45%. A H_2_O minimum detection limit of ~42 ppm was achieved at the same integration time. Measurements as long as 60 h were performed to demonstrate the capability of the sensor system to monitor atmospheric methane concentration. Our results demonstrate that a hygrometer with 2% precision of relative humidity measurement allows correct estimation and compensation of the methane signal with respect to the absolute humidity, even for several tens of hours of continuous monitoring. For prolonged measurements, continuous monitoring of QTF resonance frequency and quality factor may be required. The approach proposed by Wu et al. [30] or Rousseau et al. [31] can be easily implemented. Alternatively, the CH_4_ measurement can be automatically interrupted every hour in order to retrieve the resonance curve of the QTF via a dedicated LabVIEW-based code as such a measurement requires less than 1 min. If relevant variation of Q or f_0_ are detected, the QEPAS signal and the laser modulation frequency can be properly adjusted. The developed sensor takes advantage of high detection sensitivity, selectivity, and fast response time as well as robustness provided by the QEPAS technique empowered by the instantaneous self-calibration obtained using a low-cost and compact hygrometer, proving to be perfectly suitable for environmental monitoring applications. Thus, the combination of a methane QEPAS sensor with a hygrometer is a low-cost, low power consuming, and efficient alternative to a dual-gas QEPAS sensor without affecting the ultimate detection limit of methane when water signal compensation procedure is adopted. Further development of the proposed configuration will consist of the implementation of a compact and accurate PHT (pressure, humidity, temperature) sensor chip within the acoustic detection module structure, with the aim of improving accuracy of the absolute humidity real-time measurement in the detection volume and to further reduce the compactness of the sensor system.
